# Lifetime prevalence and correlates of smoking behavior in Iranian adults’ population; a cross-sectional study

**DOI:** 10.1186/s12889-019-7358-0

**Published:** 2019-08-06

**Authors:** Ibrahim Abdollahpour, Mohammad Ali Mansournia, Yahya Salimi, Saharnaz Nedjat

**Affiliations:** 1 Isfahan Neurosciences Research Center, Isfahan University of Medical Sciences, Isfahan, Iran; 2 Department of Epidemiology and Biostatistics, School of Public Health, Tehran University of Medical Sciences, Tehran, Iran; 3 Social Development and Health Promotion Research Center, Health institute, Kermanshah University of Medical Sciences, Kermanshah, Iran; 4 Department of Epidemiology and Biostatistics, School of Public Health, Kermanshah University of Medical Sciences, Kermanshah, Iran; 5 Department of Epidemiology and Biostatistics, School of Public Health, Tehran University of Medical Sciences, Knowledge Utilization Research Center, Tehran University of Medical Science, Tehran, Iran

**Keywords:** Population-based cross-sectional study, Cigarette, Second hand, Waterpipe, Smoking, Predictors

## Abstract

**Background:**

There are few if any reports concerning the joint use of waterpipe, cigarette and exposure to second-hand smoking in Tehran, Iran. Here, we simultaneously investigated the prevalence and predictors of smoking habits in Iranian adults.

**Methods:**

In this population-based cross-sectional study, we recruited 1057 Iranian adults between August 2013 and February 2015, in Tehran, a multi-ethnic city. Participants were selected using random digit dialing. Three separate logistic regression models were applied to estimate the adjusted odds ratios (95% CI).

**Results:**

Exposure to second-hand smoking was the most prevalent smoking type (37, 95% CI: 35–41%) followed by cigarette (23.9% (95% CI: 21–27%)) and water-pipe smoking (20.25% (95% CI: 18–23%)) in adults in Tehran. Almost 3.3 and 4.5% of adults reported three and two types of lifetime smoking behaviors, respectively. Age, sex, history of depression along with lifetime alcohol intake was the important predictors of all three types of smoking. Lifetime alcohol consumption was associated with increased prevalence of all three types of smoking (p for trend < 0.009). Lifetime drug abuse was also associated with increased prevalence of cigarette smoking (OR = 2.04, 95% CI: 1.61–2.59, *p* < 0.001).

**Conclusions:**

Lifetime prevalence of waterpipe, cigarette and exposure to second-hand smoking is moderately high. Dual smoking behaviors are increasing in Iranian adults. An apparent age-related difference in pattern of waterpipe and cigarette smoking was observed. These findings highlight the need for further educational and preventive programs especially for dual smoking in Iranian young adults. This could provide practical information for evaluating and reforming the tobacco control programs and policies in Iran.

## Introduction

Tobacco smoking is one of the main leading global causes of avoidable premature mortality [1] and still remains as one of the main global public health issues [2]. It is responsible for almost 5 million (~ 20%) of the worldwide annual deaths [3, 4]. It is expected that this number would increase to more than 8 million in 2030 [5], of which around 80% will occur in countries with low or middle-income level [6]. Its causal role in the development of adverse health related outcomes including several types of cancer [7–9], chronic diseases [5, 10] as well as its high economic cost [4, 11] has been proven already.

Waterpipe smoking, also known as hookah, is the oldest form of tobacco smoking [12]. It is not only the most common method of tobacco use among youth in the Middle East, but also it is increasingly becoming an important global tobacco use method [13, 14]. It has been shown in a number of studies that the adverse health outcome of waterpipe smoking is equal to or worse than cigarettes smoking [15]. Evidence supports a positive association between waterpipe smoking and the risk of cancer [16], respiratory and cardiovascular disease [15, 17] and multiple sclerosis [18]. The common misunderstanding about the less dangerous nature of waterpipe smoking may be responsible for the increasing prevalence of waterpipe smoking [19].

There are substantial differences between and within geographical world regions regarding the prevalence of smoking [4]. The burden of tobacco use in Iran is considerable [20]. Based on the results of a meta-analysis published in 2013, almost 20% of Iranian male adults smoke [21]. Currently, few surveys simultaneously addressed the prevalence and modifiable correlates of waterpipe smoking, cigarette smoking and exposure to second-hand smoking in Iran. Tehran, the Capital of Iran, with a wide variety of ethnics and mass linguistic groups from all over Iran has provided the opportunity of more generalizable estimates of the smoking pattern and its correlates. Here using a population-based study we tried to investigate smoking pattern and its predictors in 15–50-year-old in Iranian population.

## Material and methods

**Fig. 1:**
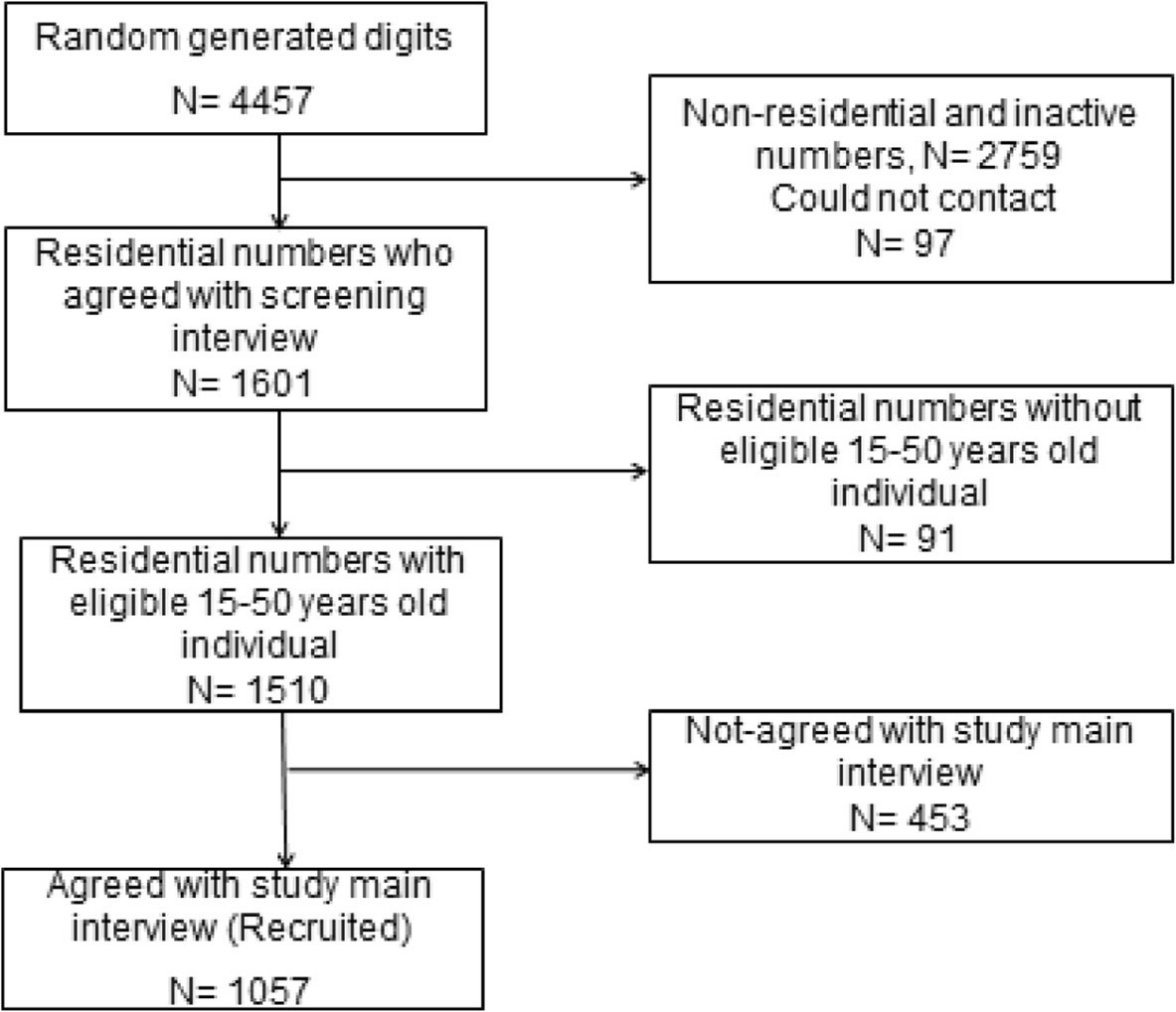
Detailed recruitment flow diagram of the study participants, Tehran, 2013–2015

In this study, we used data of a population-based cross-sectional study that was conducted between 2013 and 2015 in Tehran, Iran. [18, 22, 23]. All residents of 22 municipality areas of Tehran aged 15–50 years constituted the study reference population. Using the standard method of random digit dialing (RDD) the study sample was selected. For generating the random dialing numbers, a 4-digit number was randomly added to the well-defined pre-codes of every 22 municipality areas of Tehran. The participants were selected proportionally to population size of each 22 areas of Tehran which had been determined in the last national Iranian census report. Using the Kish method [24], we selected one eligible individual in each of the selected household. The Kish method is a detailed process in order to randomly selecting one or more individuals when randomly calling to the households. In sum, it gives priority to the male and older residents and then, based on the participants’ gender and age, gives a rank to every resident of household. Using pre-specified specific tables, the Kish method randomly selects one or more individuals among the residents of each household [24]. Detailed recruitment flow-chart of the study participants has been shown in Fig. 1. As demonstrated, 1057 (70.0%) of 1510 contactable households, within which at least one eligible person existed, have been fully interviewed. Ten trained interviewers conducted the interviews. [18]. At the start of each interview, the participants were informed about the goals of the study and oral consent was obtained. Each interview on average took 15 min.

### Data collection protocol

Ten interviewers which were selected for their strong communication and interview skills, and trained to use the standardized data collection procedures conducted the phone interviews. We monitored the data collection activities for any interviewer bias by randomly recording interviews[18]. At the start of each interview, the participants were informed about the study main objectives. Since the data collection process was conducted using phone interviews, we obtained oral instead written informed consent from all of the participants. In the case of fewer than 16 years old participants, the oral informed consent was obtained from one of their parents.

### Measurement

The participants were requested to provide all required information before the sampling date i.e. the date of recruiting in the study.

#### Smoking

Participants who had history of waterpipe smoking for at least once a week for a minimum 6 months were categorized as those with history of waterpipe smoking. We also asked detailed information on duration (years), amount (average frequency per week). We calculated the participants’ cumulative waterpipe smoking by multiplying the average frequency per week by 52 weeks and the duration (years) [18, 25]. In addition, information on cigarette smoking (total duration (years), average amount smoked per day, converted to pack-years), exposure to second-hand smoking (ever lived with anyone who regularly smoked, duration (years), timing (before/after or during 13–19 years))[26] was also obtained. We obtained the data on second-hand smoking for both of smokers and non-smokers.

#### Lifetime drug use

We measured any history of drug/substance use asking the following question “Have you ever used any type of substance for at least once monthly during at least a 6-month period?” We also collected detailed information on type of drug/substance use i.e. (opioids, cannabis, inhalants, hallucinogens and stimulants), duration (years) as well as amount (average frequency per month). The average frequency of a drug per month was multiplied by 12 and the duration (years), and then was summed for the different drug types in order to calculating the lifetime drug abuse [26].

#### Lifetime alcohol intake

Lifetime history of alcohol consumption was defined as any type of alcohol drinking at least 6 times in at least a six-month period. For 4 types of beverages (beer, wine and liquor), detailed information on lifetime alcohol intake (i.e. duration (years), average number of drinks per month and average drink size of each drink (ml)) were collected [22]. We employed the University of Minnesota’s nutrient data system for calculating total lifetime intake of ethanol (g) [27]. This system assigns 12.8, 13.8 and 14.0 g of alcohol for each 355 ml of beer, 177 ml wine, and 44 ml liquor, respectively. Life-time intake for each source of alcoholic beverage was calculated by multiplying the received gr of specific alcoholic beverage in each drink by the average number of drinks per month, which was multiplied by 12 months and the duration (years) Receivedgrofspecificalcoholicbeverageineachdrink × averagenumberofdrinkpermonth × 12 × duration(year).. Then, total lifetime alcohol consumption (g) was calculated as the sum of three lifetime specific alcoholic beverages. We applied pre-specified categories of alcohol intake for classifying study participations (never, ≤500 g, 500–5000, > 5000 g)[26].

#### History of depression

Lifetime self-reported history of depression was measured by using the following question: “Have you ever received a diagnosis of depression from a mental health professional?” [28].

#### Life event number

Data on the presence of stressful life events (SLE) was obtained asking for example; “Did you experience a serious illness happened for one of your family members?” We collected data on the following SLEs; marriage, death of spouse, death of a loved one including close relatives’ (e.g. parent or sibling), divorce, death of one of your dear ones, jail term, retirement, severe illness of family members, family disruption (divorce of the parents), suicide, conquer (national exam for university entrance), dismissal from work, migration, homeless periods, and being in debt as well[26]. The total number of stressful life events was calculated summing all 15 life events resulting in the total stress number.

#### Physical activity

Respondents were asked about their physical activity level during adolescence (frequency per week, intensity level (severe or light) and average duration (minutes))[29]. Metabolic equivalent of task (MET) was then calculated using the collected data [26, 30].

#### Demographics variables

The questionnaire also included demographic questions (age, sex, marital status and years of education,

### Statistical analysis

Logistic regression was used to estimate crude and adjusted odds ratios (ORs) (STATA 12) in 2018. Several variables were examined to detect their association with smoking types including age, sex, marital status, years of education, lifetime alcohol intake, lifetime drug use, stressful life event and history of depression. Test for trend of ordinal categorical variables were undertaken by replacing the binary predictors with a single predictor, taking category rank scores.

## Results

The detailed recruitment flow-chart of participants is shown in Fig. 1. Of the 1510 contactable households with eligible people, 1057 (70.0%) agreed to a full interview. The mean age was 31.3 and 51.5% of them were female. The majority of participants were less than 40 years old (Table 1).

**Table 1:** Characteristics of 1057 study population, Tehran, 2013–2015

Variables	*N* (%) ^a^
Female gender	544 (51.50)
Age at the time of participant (years); mean (SD)	31.3 (9.33)
Age at the time of participant (Categories)	
< 20	349 (33.05)
20 to less than 30	362 (34.28)
30 to less than 40	209 (19.79)
40–50	136 (12.88)
Marital status	
Single	462 (43.90)
Married	591 (56.10)
Highest level of education	
Illiterate or primary school (age 7–11 yrs)	26 (2.47)
Guidance school (age 12–14 yrs)	60 (5.6)
High school (age 15–18 yrs)	437 (41.34)
Associate’s or Bachelor’s degree	441 (41.72)
Master’s degree and higher	93 (8.80)
Years of education (year); mean (SD)	13.43 (3.26)

^a^ No. (%), except where otherwise indicated. In the case of missing data, the sum of categories is less than 1057

### Gender differentials in prevalence of smoking behaviors

On average males started both cigarette and waterpipe smoking sooner than females (18.95 years old vs. 22.42 years old for cigarette and 19.95 years old vs. 20.85 years old for waterpipe smoking). While a higher proportion of males smoked waterpipe and cigarette for > 5 and 10 years, the proportion of females exposed to second-hand smoke for > 10 years is higher. Total current cigarette and waterpipe use was 14.38% (95% CI (12–17%)) and 16.60% (95% CI (15–19%)) in the study population, respectively. More than 35% of males have reported the life-time experience of cigarette and waterpipe smoking. However, exposure to second-hand smoking (41%) followed by waterpipe smoking (13.1%) is the most common smoking in females (Table 2).

**Table 2:** Gender specific prevalence of different types of smoking among adults aged 15–50, Tehran, 2013–2015

Variables	Study population *N* (%)	Male	Female	Statistical test	OR (95% CI)	*P* value
Waterpipe Smoking						
Never	803 (76.10)	331 (64.65)	472 (86.92)	Simple logistic	0.27 (0.20, 0.37)	< 0.001
Ever	252 (23.90)	181 (35.35)	71 (13.08)			
Current	176 (16.70)	127 (24.80)	49 (9.02)	Simple logistic	0.27 (0.19, 0.39)	< 0.001
Past	76 (7.20)	54 (10.55)	22 (4.06)		0.28 (0.17, 0.48)	< 0.001
Age at first Waterpipe Smoking	20.30 (5.65)	19.95 (5.45)	20.85 (5.93)	Simple logistic	1.05 (1.00, 1.10)	0.039
Waterpipe Smoking – duration (years)						
≤ 5	109 (10.35)	67 (13.09)	42 (7.73)	Simple logistic	0.44 (0.29, 0.66)	< 0.001
> 5	143 (13.55)	114 (22.27)	29 (5.34)		0.18 (0.12, 0.27)	< 0.001
≤ 250	121 (11.5)	67 (13.09)	42 (7.73)	Simple logistic	1	–
> 250	127 (12.1)	114 (22.27)	29 (5.34)		0.28 (0.20, 0.38)	< 0.001
Cigarette smoking						
Never	843 (79.75)	329 (64.13)	514 (94.49)	Simple logistic	1	–
Ever	214 (20.25)	184 (35.87)	30 (5.51)		0.1 (0.07, 0.16)	< 0.001
Current	152 (14.38)	130 (25.34)	22 (4.04)	Simple logistic	0.11 (0.07, 0.17)	< 0.001
Past	62 (5.87)	54 (10.53)	8 (1.47)		0.09 (0.04, 0.20)	< 0.001
Age at first cigarette smoking	19.67 (4.71)	18.95 (4.39)	22.42 (4.95)	Simple logistic	1.13 (1.04, 1.23)	0.004
Cigarette smoking – duration (years)						
≤ 10	122 (11.50)	100 (19.49)	22 (4.04)	Simple logistic	0.14 (0.87, 0.22)	< 0.001
> 10	92 (8.70)	84 (16.37)	8 (1.47)		0.06 (0.03, 0.13)	< 0.001
Cigarette smoking (total pack-years)						
≤ 5	165 (15.60)	140 (27.40)	26 (4.78)	Simple logistic	0.11 (0.07, 0.18)	< 0.001
> 5	46 (4.40)	42 (8.22)	4 (0.73)		0.06 (0.02, 0.17)	< 0.001
Second-Hand Smoking						
Never	664 (63.00)	344 (67.32)	320 (58.93)	Simple logistic	1	–
Ever	390 (37.00)	167 (32.68)	223 (41.07)		1.43 (1.11, 1.85)	0.005
Second-Hand Smoking – duration (years)						
≤ 10	101 (9.58)	43 (8.41)	58 (10.68)	Simple logistic	1.45 (0.95, 2.12)	0.085
10–20	139 (13.19)	59 (11.55)	80 (14.73)		1.46 (1.01, 2.11)	0.045
> 20	150 (14.23)	65 (12.72)	85 (15.65)		1.43 (0.98, 2.01)	0.062
Second-Hand Smoking – age period						
Before 19 years old	170 (16.13)	83 (16.24)	87 (16.02)	Simple logistic	1.13 (0.80, 1.58)	0.488
After 20 years old	80 (7.59)	24 (4.70)	56 (10.31)		2.51 (1.52, 4.14)	< 0.001
Throughout life	140 (13.28)	60 (11.74)	80 (14.73)		1.43 (0.99, 2.07)	0.055
Drug abuse						
Never	984 (93.27)	445 (86.91)	540 (90.30)	Simple logistic	1	–
Ever	71 (6.73)	67 (13.09)	4 (0.70)		0.04 (0.01, 0.12)	< 0.001
History of depression						
Yes	170 (16.14)	55 (10.78)	115 (21.18)	Simple logistic	1	–
No	883 (83.86)	455 (89.22)	428 (78.82)		2.22 (1.57, 3.14)	< 0.001
Physical activity						
Acceptable MET/week	415 (39.52)	301 (59.14)	114 (21.07)	Simple logistic	1	–
Lower than acceptable	436 (60.48)	208 (40.86)	427 (78.93)		5.42 (4.13, 7.11)	< 0.001

### Prevalence of different smoking behaviors

Smoking is common among 15–50 adult populations. Second-hand smoke exposure (37% (95% CI: 35–41%)), waterpipe smoking (23.90% (95% CI: 21–27%)) and cigarette (20.25, 95% CI: 18–23%) smoking are the most common types of smoking respectively (Table 3).

**Table 3:** Age specific prevalence of different types of smoking among adults aged 15–50, Tehran, 2013–2015

Variables	Study population *N* (%)	< 30 years	30–50 years
Waterpipe Smoking			
Never	803 (76.10)	337 (69.77)	466 (10.5)
Lifetime	252 (23.90)	147 (30.63)	105 (18.39)
Current	176 (16.60)	120 (24.64)	56 (9.81)
Past	76 (7.20)	27 (5.59)	49 (8.58)
Age at first waterpipe Smoking	20.30 (5.65)	17.91 (3,71)	23.62 (6.30)
Waterpipe Smoking – duration (years)			
≤ 5	109 (10.35)	80 (73.040)	29 (26.60)
> 5	143 (13.55)	67 (46.85)	76 (53.15)
Waterpipe Smoking – cumulative amount (pipes)			
≤ 250	121 (11.5)	68 (56.20)	53 (43.80)
> 250	127 (12.1)	76 (59.84)	51 (40.16)
Cigarette smoking			
Never	843 (79.75)	403 (47.86)	440 (52.14)
Lifetime	214 (20.25)	82 (38.32)	132 (61.68)
Current	152 (14.38)	63 (41.45)	89 (58.55)
Past	62 (5.87)	19 (30.63)	43 (69.37)
Age at first cigarette smoking	19.67 (4.71)	17.90 (3.37)	20.34 (4.74)
Cigarette smoking – duration (years)			
≤ 10	122 (11.5)	69 (56.56)	53 (43.33)
> 10	92 (8.70)	13 (14.13)	79 (85.87)
Cigarette smoking (total pack-years)			
≤ 5	165 (15.60)	74 (44.85)	91 (55.15)
> 5	46 (4.40)	7 (15.22)	39 (84.78)
Second-Hand Smoking			
Never	664 (63.00)	306 (46.50)	358 (53.50)
Lifetime	390 (37.00)	178 (45.64)	212 (54.36)
Second-Hand Smoking – duration (years)			
≤ 10	101 (9.58)	57 (56.44)	44 (43.56)
10–20	139 (13.19)	69 (49.64)	70 (50.36)
> 20	150 (14.23)	52 (34.67)	98 (65.33)
Second-Hand Smoking – age period			
Before 19 years old	170 (16.13)	91 (53.53)	79 (46.47)
After 20 years old	80 (7.59)	29 (36.25)	51 (83.75)
Throughout life	140 (13.28)	58 (41.43)	82 (58.57)

### Joint use of different types of smoking

While, more than 42% of people did not report any smoking experience, 3.3% of them reported all three types of smoking behaviors. Moreover, 4.5% of participants reported the lifetime experience of at least two types of smoking (Table 4).

**Table 4:** The joint use of different types of smoking, Tehran, 2013–2015

Variables	General population *N* (%)
Cigarette & Waterpipe smoking^a^	
Never cigarette or waterpipe	683 (64.75)
Just cigarette	116 (11.00)
Just waterpipe	159 (15.05)
Both cigarette & waterpipe	97 (9.20)
Cigarette & second-hand^a^	
Never cigarette or exposure to second-hand	529 (50.19)
Just exposure to second-hand	312 (29.60)
Just cigarette	129 (12.24)
Both cigarette & exposure to second-hand	84 (7.97)
Waterpipe & second hand^a^	
Never waterpipe or exposure to second-hand	513 (48.67)
Just exposure to second-hand	289 (27.42)
Just waterpipe	145 (13.76)
Both waterpipe & exposure to second-hand	107 (10.15)
All three types of smoking^b^	
Never waterpipe, cigarette or exposure to second-hand	447 (42.41)
Just waterpipe	87 (8.25)
Just cigarette	71 (6.74)
Just exposure to second-hand	236 (22.39)
Waterpipe & cigarette	59 (5.60)
Waterpipe & exposure to second-hand	71 (6.74)
Exposure to second-hand & cigarette	48 (4.55)
All 3 types of smoking	35 (3.32)

^a^These are combination of every possible 2 smoking habits

^b^“All three type of smoking” is every possible combination of all 3 smoking habits

### Predictors of three types of smoking

While life-time waterpipe smoking was significantly associated with an increased prevalence of both cigarette smoking (OR = 1.65, 95%CI: 1.15–2.36, *p* = 0.007) and exposure to second-hand smoking (OR = 1.47, 95% CI: 1.12–1.91, *p* = 0.005), lifetime exposure to second-hand smoking was only associated with waterpipe smoking (OR = 1.47, 95% CI: 1.13–1.92, *p* = 0.004). Similarly, cigarette smoking increased the odds of waterpipe use (OR = 1.55, 95% CI: 1.08–2.21, *p* = 0.018) but not exposure to second-hand smoking. While increasing in age significantly increased the life time cigarette smoking, conversely it decreased the probability of waterpipe smoking. Those older than 40 years, had a four-fold decrease in the odds of waterpipe smoking (OR = 0.25, 95% CI: 0.14–0.47, *p* < 0.001). Prevalence of both cigarette and waterpipe in males were significantly higher than females. Females were more likely to expose to second-hand smoking. Lifetime drug abuse only increased the prevalence of cigarette smoking (OR = 2.04, 95% CI: 1.61–2.59, *p* < 0.001). However, lifetime alcohol consumption was associated with increased prevalence of all three types of smoking (p for trend < 0.009). Both cigarette and exposure to second-hand smoking were affected by the total number of stressful life events. Education was inversely associated with only exposure to second-hand smoking (OR = 0.92, 95% CI: 0.89–0.95, *p* < 0.001). The level of physical activity was lower than acceptable of 60.48% of the study participants. However, the physical activity as well as marriage status was not associated with any types of smoking (data not shown). Finally, the depression history significantly predicted all three smoking with the strongest association with cigarette smoking (OR = 1.81, 95% CI (1.22–2.70)) followed by waterpipe smoking and second hand-smoking (Table 5).

**Table 5:** Three multivariable models demonstrating predictors of three types of smoking habits, Tehran, 2013–2015

Predictors	Cigarette Smoking		Waterpipe Smoking		Exposure to Second-Hand Smoking	
	Adjusted OR (95% CI)	*P*-value	Adjusted OR (95% CI)	*P*-value	Adjusted OR (95% CI)	*P*-value
Lifetime waterpipe smoking	1.65 (1.15–2.36)	0.007	–		1.47 (1.12–1.91)	0.005
Lifetime exposure to second hand smoking	1.19 (0.85–1.66)	0.322	1.47 (1.13–1.92)	0.004	–	
Cigarette Smoking (Lifetime vs. never)	–		1.55 (1.08–2.21)	0.018	1.08 (0.77–1.50)	0.663
Lifetime drug use (cumulative number)						
Never	1		1		1	–
1–50	6.67 (2.52–17.64)	< 0.001	1.70 (0.76–4.22)	0.229	1.15 (0.53–2.50)	0.716
51–500	2.98 (1.26–7.07)	0.013	1.64 (0.81–3.81)	0.219	0.76 (0.38–1.53)	0.444
> 500	7.84 (3.45–17.85)	< 0.001	0.75 (0.42–1.48)	0.380	1.15 (0.64–2.09)	0.632
Test for trend	2.04 (1.61–2.59)	< 0.001	0.99 (0.81–1.20)	0.880	1.01 (0.85–1.21)	0.881
Lifetime alcohol intake – cumulative amount (g)						
Never			1		1	–
≤ 500	4.52 (2.80–7.29)	< 0.001	2.86 (1.86–4.40)	< 0.001	1.22 (0.81–1.84)	0.334
500–5000	4.54 (2.77–7.46)	< 0.001	3.93 (2.50–6.18)	< 0.001	1.33 (0.86–2.05)	0.196
> 5000	5.71 (3.49–9.34)	< 0.001	3.55 (2.23–5.67)	< 0.001	1.76 (1.14–2.72)	0.011
Test for trend	1.88 (1.61–2.18)	< 0.001	1.65 (1.43–1.91)	< 0.001	1.19 (1.04–1.37)	0.009
History of depression	1.81 (1.22–2.70)	0.003	1.78 (1.30–2.44)	< 0.001	1.46 (1.14–1.89)	0.003
Categorized life event number						
0	1		1		1	–
1–2	1.77 (1.08–2.88)	0.022	1.05 (0.74–1.52)	0.773	1.32 (0.99–1.76)	0.059
> 3	1.94 (1.15–3.29)	0.013	1.28 (0.85–1.91)	0.230	1.99 (1.44–2.75)	< 0.001
Test for trend	1.32 (1.04–1.69)	0.024	1.14 (0.94–1.39)	0.181	1.42 (1.21–1.67)	< 0.001
Age						
> 20	1		1		1	–
20–29.999	3.26 (1.51–7.03)	0.003	1.70 (1.05–2.74)	0.029	1.31 (1.04–2.60)	0.173
30–39.999	3.77 (1.75–8.13)	0.001	0.86 (0.53–1.41)	0.557	1.10 (0.52–1.36)	0.646
40–50	5.27 (2.37–11.72)	< 0.000	0.25 (0.14–0.47)	< 0.001	1.26 (0.75–1.74)	0.293
Male sex	6.01 (3.12–8.77)	< 0.001	2.47 (1.83–3.34)	< 0.001	0.54 (0.42–0.70)	< 0.001
Years of education	0.96 (0.91–1.01)	0.122	0.99 (0.94–1.03)	0.536	0.92 (0.89–0.95)	< 0.001

## Discussion

Using population-based data in Iranian adults, we found exposure to second-hand smoking as the most prevalent smoking behavior (37%) followed by water-pipe (23.9%) and cigarette smoking (20.25%), respectively**.** Age, sex, history of depression along with lifetime alcohol intake were the important predictors of all three types of smoking habits. Moreover, cumulative life-event number predicted the cigarette and exposure to second-hand smoking. The lifetime drug abuse was only associated with cigarette smoking. The latest statistics provided by Drope et al. in Tobacco atlas demonstrated that 14.2% of males and 0.4% of females smoke cigarettes daily [31]. This is less than our finding (41.07% in females vs. 32.68% in males) in Tehran.

We identified exposure to second-hand smoking as the most prevalent smoking type in adults of Tehran. This finding indicated that current public places smoke-free policies may not be effective enough. Moreover, it emphasizes the need for strengthening health-related knowledge and also planning effective health education programs in family environments. In a cross-sectional study enrolled 5900 adults (15–75 years old) in Kerman, the prevalence of exposure to second-hand smoking was 27.5% (30.1% in females vs. 25.0% in males) [32] which is moderately less than our findings (41.07% in females vs. 32.68% in males). Our estimate is moderately less than one published systematic review by Zeng et al. in 2016 in which the prevalence of exposure to second-hand smoking in Chinese 15–59 years old was 47.1 [33].

In a cross-sectional study in Tehran which recruited 1830 participants, the prevalence of current waterpipe smoking in ≥15 years old was 17.6% which is similar to our estimates [34]. However, the lifetime prevalence of waterpipe smoking in current study was higher than recent estimates (12%) for the UK in the 11–16 years old age group [35]. The different recruited age group of the UK study potentially limits the comparability of findings. In California Cigarette Survey study by Smith et.al, 24.5% of young men (18–24) were ever waterpipe smokers [36]. In a systematic review conducted in 2008 in Middle Eastern countries, the minimum and maximum national reported prevalence of current waterpipe smoking among adults were 5 and 15%, respectively[13]. While, evidence shows that the prevalence of cigarette smoking is decreasing in developed countries, an observed increasing prevalence of the other forms of tobacco smoking such as waterpipe smoking is highly concerning [36, 37]. A most common public misunderstanding of the less harmful adverse health effects of waterpipe compared to cigarette smoking may be one of the possible reasons for this increasing prevalence [38]. Nonetheless, studies have shown that sweetened flavored tobacco products used in waterpipe, contain several toxicant agents, carcinogenic materials and heavy metals [39, 40]. Moreover, the carbon monoxide dose as well as the volume of inhaled smoke when smoking waterpipe are very much higher than those in cigarette because of exposure to the charcoal used for heating the waterpipe [15]. There is also evidence demonstrating that wastepipes have adverse short-term and long-term health effects including infectious, respiratory and cardiovascular disease, multiple sclerosis, cancer as well as mental health disorders leading to an increased risk of illegal substance use in young people risk [17, 41].

In a population-based cross-sectional study in Tehran by Fotouhi et al. which enrolled 3397 residents, the reported prevalence of smoking (11.9%) was less than our estimate (20.25%) [42]. This could suggest an increasing trend in cigarette smoking among 15–50 years old residents of Tehran. The lifetime prevalence of cigarette smoking in males in this study is higher than results of a published meta-analysis in 2014 which reported that nearly 25% of 15–65 years old males of northern Iran were ever smokers [43]. Our estimate is also more than a published meta-analysis in 2013 in Iran in which only 20% of Iranian male adults have reported history of cigarette smoking [21]. The prevalence of three types of smoking habits in males and females were higher than those reported in an Iranian national report by Nemati et.al study. The results of this national report showed that 10.9 and 2.4% of the Iranian adults were daily cigarette and waterpipe smoker, respectively [44]. Moreover, the estimated joint prevalence of cigarette and waterpipe smoking in the present study was substantially higher than those reported in the Iranian national report by Nemati et.al (5.6% vs. 0.3%) [44].

The estimated prevalence of cigarette smoking in the current study is comparable with finding of a meta-analyses among Iranian adults published in 2013 [21]. In recent meta-analysis the range of cigarette smoking was reported between 12.3 to 38.5% in men, and between 0.6 to 9.8% in women, which is comparable to our estimates [21].

Consistent with a study by Albisser et al. conducted in Switzerland among 204 young adults [45], our findings indicated that waterpipe smoking could predict both cigarette and exposure to second-hand smoking habits. Although exposure to second-hand and cigarette smoking were associated with each other, they could not predict engaging in the water pipe smoking.

Alcohol intake was highly correlated with both lifetime waterpipe and cigarette smoking (p for trend< 0.05). Consistently, Tamim et al., in a cross-sectional study recruited 1964 students from Lebanon Universities, found that heavy drinkers were more likely to be waterpipe and cigarette smoker [46]. It is well-acknowledged that cigarette smoking can predispose to alcohol and substance use. The possible mechanism of this association is still not clear. As expected, engaging in drug use activities was associated with increased likelihood of cigarette smoking [47]. However, the cross-sectional nature of this design limits the proper interpretation of the temporality between cigarette smoking and drug abuse. Depression was the other factor that predicted waterpipe and cigarette smoking habits but not exposure to second-hand smoking. This was similar to the other studies [48, 49], but not all of them [50]. The cumulative number of life time stressful events was associated with both cigarette and exposure to second-hand but not waterpipe smoking (p for trend< 0.05). Prior studies have similarly demonstrated that the higher level of stress could directly or indirectly elevate substance use among young people [51, 52]. Further examination is needed to understand the role of life time stressful events in the context of other forms of tobacco use.

There is a gender difference in the lifetime prevalence of waterpipe (35.3% in males vs. 13.1 in females) as well as the lifetime prevalence of cigarette smoking (35.9 in males vs. 5.5% in females) in this study. In general, other than exposure to second-hand smoking, the estimated lifetime prevalence was higher in males than females. This is in line with the results of other studies [44, 53]. However, exposure to second-hand smoking was more likely in Iranian females. The later finding shows that women are often affected by the others’ smoking behaviors. Contrary to our finding, Riachy et al. in a study, recruited 37,579 participants during 2003 to 2005 in Lebanon, showed that females consumed more waterpipe than males [54]. While the likelihood of cigarette smoking increased with age, reversely, the probability of being waterpipe smoker increased in young adults (20 to 29 years old). This could well demonstrate the age-related differences in pattern of smoking behaviors in Iranian population. Finally, increased years of education could decrease the probability of exposure to the second-hand smoking in the study population.

There are some advantages and limitations that should be considered. This study recruited a population-based data set to describe lifetime prevalence and related factors of waterpipe, cigarette and exposure to second-hand smoking among Iranian 15–50 years old adults. The response rate (70%) in this study was found to be satisfactory. Although the usefulness and efficacy of RDD sampling as well as its similarity with address-based sampling were previously demonstrated [55–57], some possibility of selection bias may still have remained. This could affect the validity of study findings. Although we conducted a population-based study in a multiethnic city, the generalizability of the study finding to the whole Iranian population should be done after considering the rural vs. metropolitan differences in smoking behavior. The more stressful nature of metropolitan environments [58], increasing smoking habits in immigrate of the metropolitan areas [59], and finally negative attitude toward smoking habits in the rural areas may differentiate the prevalence of smoking habits in rural area compared to urban area. Considering the illegal nature of alcohol and illicit drugs consumption in Iran society, there was the possibility of the underreporting with respect to these sensitive behaviors. However, we reported one of the largest estimates of the three smoking behaviors as well as alcohol intake and drug abuse compared to the previous studies in Iran. This could alleviate the possibility of a major under reporting. Moreover, the cross-sectional nature of the study design, especially the temporality issue, precluded us from drawing causal conclusions with respect to the identified associations.

## Conclusions

Lifetime prevalence of waterpipe, cigarette and exposure to second-hand smoking is moderately high among Iranian adults. Similarly, compared with previous studies, dual smoking behaviors are increasing in Iranian adults. Age and male sex, history of depression, life time stressful events, drug abuse, alcohol consumption as well as years of education were all found to have moderate correlations with smoking habits. There was an apparent age-related difference in pattern of waterpipe and cigarette smoking behavior in Iranian population. These findings highlight the need for further educational and preventive programs, especially for dual smoking in Iranian young adults, to stress the underlying potential adverse effects of waterpipe smoking. This could provide practical information for evaluating and reforming the tobacco control programs and policies in Iran.

## Data Availability

The datasets used and/or analyzed during the current study are available from the corresponding author on reasonable request.

## Abbreviations

MET: Metabolic equivalent of task
OR: Odds ratios
RDD: Random digit dialing
SLE: Stressful life events
